# Magnetization Transfer and Amide Proton Transfer MRI of Neonatal Brain Development

**DOI:** 10.1155/2016/3052723

**Published:** 2016-11-03

**Authors:** Yang Zheng, Xiaoming Wang, Xuna Zhao

**Affiliations:** ^1^Department of Radiology, Shengjing Hospital of China Medical University, No. 36, Sanhao Street, Heping District, Shenyang 110004, China; ^2^Division of MR Research, Department of Radiology, Johns Hopkins University, Baltimore, MD, USA; ^3^Philips Healthcare, Beijing, China

## Abstract

*Purpose.* This study aims to evaluate the process of brain development in neonates using combined amide proton transfer (APT) imaging and conventional magnetization transfer (MT) imaging.* Materials and Methods.* Case data were reviewed for all patients hospitalized in our institution's neonatal ward. Patients underwent APT and MT imaging (a single protocol) immediately following the routine MR examination. Single-slice APT/MT axial imaging was performed at the level of the basal ganglia. APT and MT ratio (MTR) measurements were performed in multiple brain regions of interest (ROIs). Data was statistically analyzed in order to assess for significant differences between the different regions of the brain or correlation with patient gestational age.* Results.* A total of 38 neonates were included in the study, with ages ranging from 27 to 41 weeks' corrected gestational age. There were statistically significant differences in both APT and MTR measurements between the frontal lobes, basal ganglia, and occipital lobes (APT: frontal lobe versus occipital lobe *P* = 0.031 and other groups *P* = 0.00; MTR: frontal lobe versus occipital lobe *P* = 0.034 and other groups *P* = 0.00). Furthermore, APT and MTR in above brain regions exhibited positive linear correlations with patient gestational age.* Conclusions.* APT/MT imaging can provide valuable information about the process of the neonatal brain development at the molecular level.

## 1. Introduction

Brain development is a gradual process of continuous maturation, with varying degrees of brain maturity associated with each different developmental period. Maturation of the neonatal and infant brain is a rapid process, occurring predominantly from five months gestational age to approximately one year after birth. During this period, maturation of the developing brain primarily involves neuronal myelination, accomplished via oligodendroglial cell proliferation. This neuroglial cell proliferation is characterized by the synthesis of various proteins, leading to increased total protein content of developing brain tissue.

However, the process of myelination is not homogeneous, generally progressing from caudal to rostral, central to peripheral, and dorsal to ventral [1–5]. Consequently, the different regions of the brain exhibit concurrent differences at the molecular level across each stage of development [6]. Concordantly, the regional progression of myelination produces corresponding differences in appearance upon imaging of the developing brain, exhibiting a consistent pattern of changes on conventional magnetic resonance imaging (MRI) sequences over the course of maturation.

With the development and wide availability of MR, the diffusion tensor imaging (DTI) is used to evaluate the neonatal brain development more frequently, especially in showing the structure of white matter fiber with obvious advantages. DTI can quantify and display the diffusion properties of water molecule in brain tissues, and it is usually used to display white matter microstructure [7, 8]. Magnetic resonance spectroscopy (MRS) is also applied for evaluation of brain development. In ^1^H-MRS, the changes of N-acetylaspartate (NAA), Creatine (Cr), Choline (Cho), myo-Inositol (mI), glutamate (Glu), glutamine (Gln), Glu + Gln (Glx), and so forth in the neonatal period with gestational age/region can be observed [9, 10]. ^31^P-MRS can evaluate brain development from the perspective of energy metabolism with changes of phosphomonoesters (PME), phosphodiesters (PDE), phosphocreatine (PCr), and so forth [11]. DTI and MRS can evaluate the brain development process from the point of white matter microstructure and cerebral metabolic substance, while some proteins, cholesterol, and lipids are also associated with the brain development. Magnetization transfer (MT) and amide proton transfer (APT) imaging are sensitive to semisolid macromolecules (cholesterol and some lipids) and proteins, and so they can help to detect the changes of the above substances during neonatal brain development period at molecular level.

MT is an MRI technique that offers the ability to quantify structural differences in the central nervous system (CNS). MT imaging contrast is achieved through interactions between protons bound to semisolid macromolecules and the free water protons of biological tissue [12]. MT-MRI utilizes a radiofrequency (RF) pulse applied only to the protons of the semisolid macromolecules, which become saturated. The tightly bound protons in the macromolecular pool then undergo transfer of saturation to water, which modulates MR signal. The effects of MT in tissue can be quantified by calculating the MT ratio (MTR), which indicates the percentage of full or partial MR signals generated by saturation of the biological macromolecules. Predictably, the molecular differences between various tissues yield correspondingly divergent MTR values. In the brain [13], the primary semisolid macromolecular determinants of measured MTR values include cholesterol and other lipids.

APT imaging, a newer technique derived from MT imaging, accomplishes molecular MR imaging based on the principles of CEST [14, 15]. APT imaging generates tissue contrast through the in vivo detection and quantification of endogenous free proteins and polypeptide chains in tissues. Relatively higher APT signals generally indicate elevated exchange rates resulting from increased protein concentration [16, 17]. Although the clinical applications of APT imaging remain in their investigative stages, this modality has demonstrated promise for the evaluation of brain development [18] and the characterization and grading of brain tumors [19–23].

Thus, the purpose of this study was to investigate the relationship between APT and MT signal and gestational age during neonatal brain development in order to further elucidate the complex mechanisms of maturation during this period of brain development.

## 2. Materials and Methods

### 2.1. Patient Population

Case data were reviewed for all patients hospitalized in our institution's neonatal ward between December 2013 and June 2014. The patient population of this study were hospitalized for the reasons of respiratory tract infections, fever, skin infections, and diarrhea. Clinically, these reasons may result in brain lesions. When clinicians suspect that there may be brain lesion, a head MR examination should be ordered. We screened the neonates without nervous system disease for further study. Exclusion criteria included history of brain abnormality established prenatally, birth asphyxia, congenital malformations of the brain, mental retardation, and other diseases of the central nervous system. No intravenous contrast was administered for any portion of the MRI examinations. This study was approved by the local Ethical Committee (ethical approval code: 2013PS280K). We obtained informed consent from the patients' guardians and permission from their primary clinicians based on clinical status before additional APT/MT imaging. Conventional MRI examination is followed by APT/MT imaging where sedation has to be used only once for the same patient. The sedative is chloral hydrate with high security for newborns.

### 2.2. Conventional MRI Examination

Patients underwent sedation prior to MRI using a 5% chloral hydrate (50 mg/kg) enema administered by an anesthesiologist 30 minutes prior to the study and were monitored by the clinically responsible physician throughout the examination. All examinations were performed on a Philips 3.0 Tesla (3 T) MRI system with pencil beam (pencil beam is a kind of B_0_ shimming method through a pencil beam volume shimming algorithm) and second-order shimming (Achieva 3T TX; Philips Healthcare Systems, Best, Netherlands), using a body coil for transmission and an eight-channel sensitivity-encoding (SENSE) receiver coil. Each examination was interpreted separately by two experienced radiologists.

The conventional brain MRI examination included T1WI, T2WI, and DWI sequences. A fast-field echo (FFE) sequence was performed for T1WI using the following parameters: TR of 200 ms; TE of 2.3 ms; FOV of 180 × 161 mm^2^; matrix of 224 × 162; slice thickness of 5 mm. Parameters for the turbo spin-echo (TSE) sequence used for T2WI were as follows: TR of 4.6 ms; TE of 200 ms; FOV of 180 × 155 mm^2^; matrix of 224 × 162; slice thickness of 5 mm. Parameters for the spin-echo (SE) sequence used for DWI were as follows: TR of 2500 ms; TE of shortest time; FOV of 200 × 200 mm^2^; matrix of 124 × 124; slice thickness of 5 mm.

### 2.3. APT- and MT-MRI Examination

#### 2.3.1. APT/MT Image Acquisition

We used a single protocol that could be processed to generate both APT and MT images simultaneously. We used the raw data to calculate both APT and MTR values. The listed parameters apply to both imaging techniques.

Axial T1WI was used for positioning of the neonates at the level of the basal ganglia prior to image acquisition. The APT/MT imaging protocol was tailored to minimize local magnetic field (B_0_) inhomogeneity and optimize signal noise ratio (SNR) while maintaining an acceptable duration of scanning time for clinical applications. The protocol selected for APT/MT imaging employed an RF saturation time of 500 ms [24] (the maximum permitted by the body coil used for the examinations).

TSE with a turbo factor (TF) of 38 was used for single-slice acquisition. A multiacquisition method with multiple RF pulses was performed to enhance SNR for MT and APT and included eight acquisitions at ±3.5 ppm offset from water frequency [25]. Image acquisition utilized the following parameters: TR of 4000 ms; TE of 8.1 ms; matrix of 108 × 71; FOV of 170 × 145 mm; slice thickness of 5 mm; SENSE factor of 2, and scan time of 4 min, 16 s.

As mentioned above, APT/MT imaging included multiple acquisitions with multiple RF pulses. Over the course of the acquisition process, the images were obtained using different frequency offsets from water. The specific selected frequency offsets are as follows (parentheses indicate multiple acquisitions and the number performed): 0, ±0.25, ±0.5, ±0.75, ±1, ±1.5, ±2, ±2.5, ±3 (2), ±3.25 (4), ±3.5 (8), ±3.75 (4), ±4 (2), ±4.5, ±5, ±6 ppm, and 15.6 ppm. An unsaturated image was used to normalize the signal.

#### 2.3.2. APT/MT Postprocessing and Data Analysis

For the ATP analysis, raw data from the image acquisition was imported to an interactive data language program (IDL; Research Systems, Inc., Boulder, CO, USA) used for data analyses and reconstruction of pseudo-color images. This software was used to calculate a voxel-based Z spectrum. A 12th-order polynomial was then employed to fit the entire Z spectrum and identify the point of lowest intensity on the Z spectrum. This information was used to characterize the inhomogeneity of the B_0_ field and subsequently to obtain field correction of the Z spectrum. The corrected Z spectrum data were applied to the MTR asymmetry (MTRasym) analysis using symmetrical ±3.5 ppm offset data points. Finally, APT images were generated from the MTRasym values that were calculated at the selected offsets using the following equation: MTRasym (3.5 ppm) = *S*_sat_/*S*_0_ (−3.5 ppm) − *S*_sat_/*S*_0_ (3.5 ppm), where *S*_sat_/*S*_0_ represents the ratio of signals obtained with (*S*_sat_) and without (*S*_0_) saturation. The measured APT values were used to reflect the relative magnitude of APT-weighted effects on generated images.

With the same technique and similar method, the MTR was defined according to the equation: MTR = 1 − *S*_sat_/*S*_0_. The measured MT spectra (plotted as a function of saturation frequency offset, relative to water) were corrected for B_0_ field heterogeneity effects on a pixel-by-pixel basis. Conventional MTR images were calculated from the saturated images at a selected offset of 15.6 ppm [25].

### 2.4. Selection of ROIs and Image Analysis

**Figure 1:**
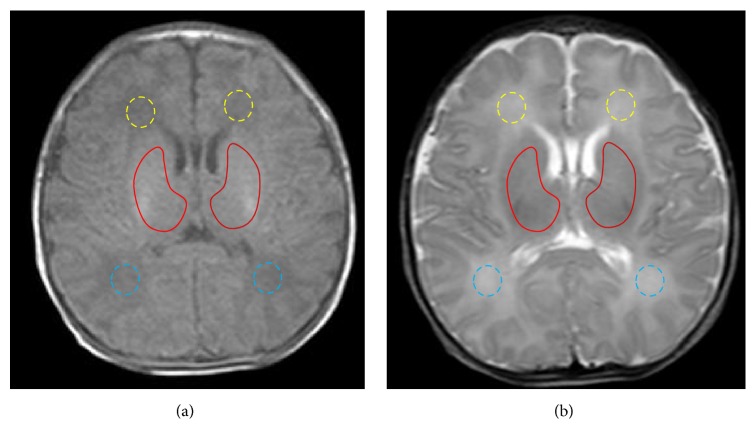
ROIs selection. Images from T1WI (a) and T2WI (b) sequences in the conventional MRI examination are referenced for the selection of ROIs in this study. For all neonates, ROIs are chosen bilaterally in the frontal lobe deep white matter (yellow dotted line), basal ganglia (solid red), and occipital lobe white matter (blue dotted line) bilaterally.

Following automated analysis of the raw acquisition data, APT and MT images generated by the software were comparatively analyzed by both senior diagnostic radiologists. With T1WI and T2WI images used as references, the process of image analysis began with the selection of ROIs. For all neonates in the study population, the ROIs included deep white matter in both frontal lobes, bilateral basal ganglia, and deep white matter in both occipital lobes (Figure 1). For ROIs selection, attempts were made to exclude the skull and cerebrospinal fluid (including the cerebral ventricles) in order to avoid associated signal interference.

Each ROI was carefully defined using the drawing function on the clinical workstation; the APT and MT values measured within the ROIs were recorded. The mapping process was performed three times for each ROI to yield the average APT/MT values. The magnitude of APT/MT values measured in the regions of acquisition was used to reflect the relative signal intensity of the ROIs on APT and MT images, with higher values manifesting increased signal intensity.

### 2.5. Statistical Analysis

Statistical analyses were performed using SPSS for Windows (Version 17.0, Chicago, IL). Quantitative data were reported as mean ± standard deviation ($\overset{-}{X}\pm S$). *P* < 0.05 was interpreted as statistically significant.

Independent two-sample *t*-test was employed to assess for significant differences in APT/MT values measured between ROIs on the left and right side at the same level of the brain. In the absence of significant differences between measurements in each hemisphere, values for each side were averaged by region and recorded for further analysis. APT and MTR values measured in each region (frontal lobe, basal ganglia, and occipital lobe) were analyzed for correlation with gestational age (in days) via Pearson's correlation analysis. ANOVA was applied to assess for statistically significant differences between APT and MTR values individually measured in frontal lobe deep white matter, basal ganglia, and occipital lobe deep white matter.

## 3. Results

### 3.1. Patient Population

A total of 38 neonates without brain abnormalities were included in the study. The patient population included both preterm and full-term infants, with corrected gestational age ranging from 27 to 41 weeks and median gestational age of 36 weeks ± 4 days.

### 3.2. APT and MT by Hemisphere and Brain Region

For all the neonates, both APT and MTR showed significant differences between the regions. The measured APT and MTR values were highest in the basal ganglia, followed by the occipital lobe and lowest in the frontal lobe (Tables 1 and 2): APT: frontal lobe white matter mean ± SD = 0.70 ± 0.29, basal ganglia mean ± SD = 1.30 ± 0.31, and occipital lobe white matter mean ± SD = 0.86 ± 0.32 and frontal lobe versus occipital lobe *P* = 0.031, frontal lobe versus basal ganglia *P* = 0.00, and basal ganglia versus occipital lobe *P* = 0.00. MTR represents frontal lobe white matter mean ± SD = 12.09 ± 1.28, basal ganglia mean ± SD = 18.16 ± 2.34, and occipital lobe white matter mean ± SD = 12.90 ± 1.09 and frontal lobe versus occipital lobe *P* = 0.034, frontal lobe versus basal ganglia *P* = 0.00, and basal ganglia versus occipital lobe *P* = 0.00.

### 3.3. APT/MT and Gestational Age

**Figure 2:**
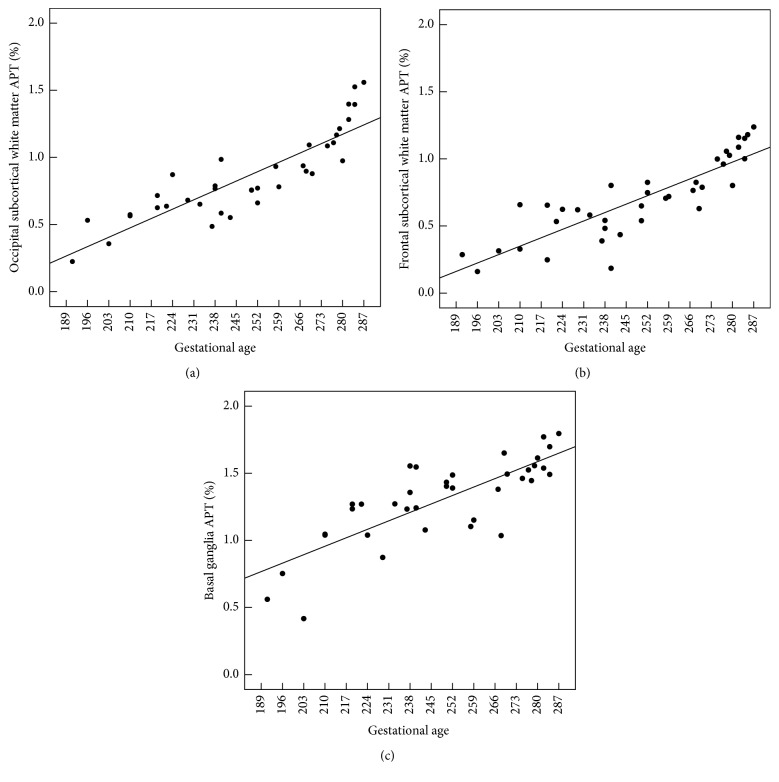
Correlations between gestational age (days) and APT values in the deep white matter of the occipital lobe (a), deep white matter of the frontal lobe (b), and basal ganglia (c). *R* ^2^: occipital lobe deep white matter: 0.75; frontal lobe deep white matter: 0.73; basal ganglia: 0.63.

APT values measured at all three regions exhibited a linear, positive correlation with gestational age (Figure 2). The strengths of correlation (reported as correlation coefficients) between APT values and gestational age observed in the three regions were as follows (in descending order): occipital lobe white matter (*r* = 0.87), frontal lobe white matter (*r* = 0.85), and basal ganglia (*r* = 0.80).

**Figure 3:**
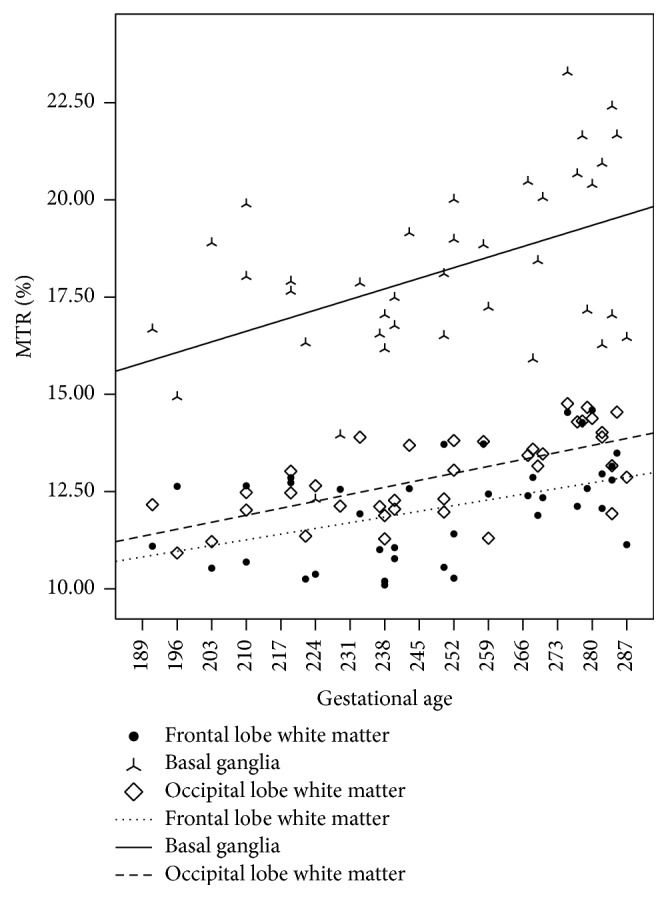
Correlations between MTR values in the brain and patient gestational age. The thin dotted line, the solid line, and the bold dotted line reflect MTR measurements in the frontal lobe white matter, basal ganglia, and occipital lobe white matter, respectively. *R* ^2^ values are as follows: occipital lobe: 0.43, frontal lobe: 0.21, and basal ganglia: 0.21.

We observed the MTR measurements (Table 2) in all three regions exhibited a linear, positive correlation with gestational age (Figure 3). Correlation coefficients observed in the three regions were as follows (in descending order): occipital lobe white matter (0.66), frontal lobe white matter (0.46), and basal ganglia (0.46).

**Figure 4:**
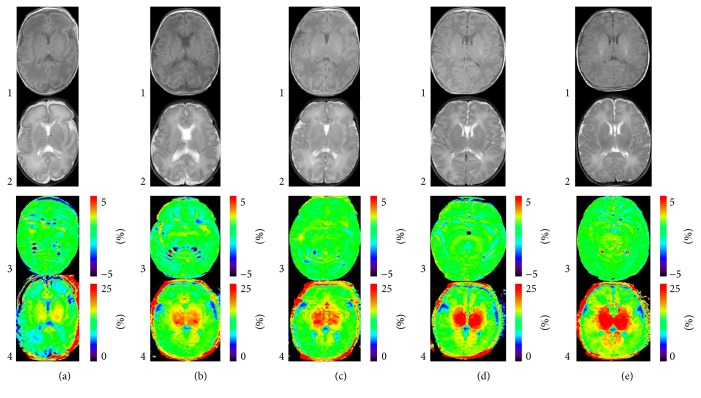
Axial images from examples of neonatal brain MRI at different corrected gestational ages. Columns (a)–(e) represent images from neonates with corrected gestational ages of 28 w, 33 w + 2 d, 35 w + 5, 37 w, and 40 w + 3 d, respectively. Images from the 4 rows are as follows: row 1 = T1WI; row 2 = T2WI images; row 3 = APT images; and row 4 = MTR images. From Figure 4, we can conclude that, with increased growth associated with age, the T2WI hyperintensity region of deep white matter showes decreased T2 hyperintensity; the formation of myelin in the posterior limb of the internal capsule is revealed as T2WI hypointensity and T1WI hyperintensity. The APT signal appears to gradually increase (signal is somewhat obscured by image contrast). Bright and dark regions (artifacts from cerebrospinal fluid (CSF)) can be seen around bilateral lateral ventricles and sulci. These scattered signals decrease with the increasing gestational age. Because with the myelination and proliferation of glial cells, the water content of brain tissue decreases, the sulci of brain becomes narrow, and the signal interference of CSF decreases. The MT signal is increased with gestational ages.

Figure 4 shows the T1WI, T2WI, APT, and MTR images of neonates with different gestational ages and shows the changes in MR during brain development.

## 4. Discussion

Initially proposed by Wolff and Balaban in 1989 [26], MT-MRI has since earned frequent application for the evaluation of brain and muscular tissue. Many prior studies have demonstrated the efficacy of MT-MRI in reflecting both the density and extent of myelination in the CNS [27–32]. APT, a newer MRI technique based on MT, generates contrast through the exchange between amide protons and water protons in order to reflect the protein content of tissues. The structural and molecular information generated by these MRI techniques offers promise in the study of the brain development. The purpose of this study was to examine the changes in different parts of the neonatal brain with increasing gestational age using MT and APT imaging.

Normally the components of the brain's internal environment and its physical and chemical properties are vital to the survival of brain cells and brain development [33, 34]. However, the neonatal brain constitutes a dynamic environment with continuous substance exchange within its internal environment. The process of neonatal brain development manifests as neuroglial cell proliferation and myelination. Neuroglial cell proliferation is observed as an increase in cell density accompanied by the synthesis of proteins for myelination [2, 3, 35, 36]. Neuroglial cell proliferation both precedes and contributes to myelination, providing both essential proteins and cytoplasmic granules containing myelin lipid precursors. Consequently, the process of myelination is associated with continuously increasing protein content in the brain during the course of development and maturation until stabilizing in adulthood.

The progressive and vigorous increase in cell density and protein content during neonatal brain maturation produces highly variable water content and complex biochemical changes in the developing brain tissue, in stark contrast to the comparative stability of adult brain tissue [3]. This dynamic biochemical environment can complicate and often confound the diagnostic evaluation of immature brain tissue on conventional MRI secondary to different signal characteristics compared to those in mature adult brains. However, the consistency of cell proliferation and protein synthesis in the developing brain offers the potential for improved characterization using newer MRI techniques such as MT and APT imaging, which generate contrast through the identification of semisolid macromolecules and proteins (both free proteins and polypeptide chains), respectively. Higher MT and APT values measured in these images reflect relatively higher semisolid macromolecular and protein content. The structural and molecular information provided by MT and APT imaging offers a potentially valuable supplement to conventional MRI examinations in the assessment of brain development.

Our results demonstrated linear, positive correlation between APT and MT values measured in different regions of the brain and patient gestational age, consistent with known physiological changes during CNS development, namely, neuroglial cell proliferation and myelination [37]. The observed correlation between MTR values and patient age in the current study is consistent with those reported in prior investigations [29, 32]. As showed in Figure 3, the *R* ^2^ values were relative small compared to the APT findings. This is due to the fact that brain development is a gradual process of myelination and glial cells proliferation. In the brain, MT effect primarily originates from semisolid macromolecular including cholesterol and other lipids [13, 32, 38], which are important components of myelin sheath. So, MT is often used to evaluate the degree of myelination, while ATP imaging can primarily detect endogenous mobile proteins (such as those dissolved in the cytoplasm) [16], which can reflect the increase of protein content in the process of glial cells proliferation and the formation of myelin sheath. The observation substances of the two imaging methods (MT and APT) are different, and that may be the reason why the correlation between MTR and gestational age is different from that of APT.

APT and MT measurements both showed significant differences between the three brain regions evaluated in this study. These regional disparities, however, can be explained by the asymmetric regional progression of neonatal brain maturation with associated underlying differences in tissue composition and inhomogeneity of local internal environments. The basal ganglia, which are rich in perikaryons and dendrites, develop earlier than the frontal and occipital lobes. Moreover, the deep white matter of the occipital and frontal lobes is composed of nerve fibers that are unmyelinated at birth. Furthermore, myelination of white matter occurs later in the frontal lobe than in the occipital lobe. Mean APT and MT values in the current study measured highest in the basal ganglia, followed by the occipital lobe deep white matter, and lowest in the frontal lobe deep white matter. This observed pattern is concordant with the demonstrated directional pattern of myelination [39–43], shown to progress from caudal to rostral, central to peripheral, and dorsal to ventral.

Zhang et al. have also recently performed an imaging study of brain development [18]. They evaluated the use of APT and MT-MRI in the characterization of pediatric brain development and observed that the APT signal in the brain decreased with increasing patient age. Although the ages of the participants in their study ranged from 0 to 16 years of age, no neonates were included in the study population, and only 16 participants were between the ages of 0 and 2 years. Consequently, the observed signal changes characterize a patient population at a later stage of the maturation spectrum than that of the current study, with more advanced degrees of myelination. In contrast, our study evaluated APT/MT signal changes within patients between 27 and 41 weeks' gestational age. The inhomogeneous biochemical and physiological patterns of maturation across different age ranges could feasibly manifest similarly different trends of APT signal change over time.

Despite the promising results of our study, several technical and performance issues must be addressed prior to routine clinical application of the APT imaging. For example, issues that limit APT/MT in its current state include eliminating direct water saturation effect, increasing the SNR, and improving image contrast [44, 45]. Safety concerns also warrant consideration, in regard to the specific absorption rate (SAR) of radiofrequency energy and the trade-off with acceptable scan time, saturation power, and flip angle.

There were several limitations in our study regarding the use of APT/MT imaging for evaluation of neonatal brain development. Firstly, our investigation is limited by the technical limitations of our available APT/MT imaging sequence, which permits only single-slice imaging. Thus, for the axial basal ganglia level selected in this study, other areas and structures were neither shown nor evaluated. Also, our study was limited to some degree by the somewhat small size of the patient population. Theoretically, the small number of patients within each gestational age group could have fostered slight bias in measured correlations between APT/MT signals and different gestational ages. However, we do not expect that the size of the patient population group should have affected the trend of APT/MT measurements across the different gestational ages.

## 5. Conclusions

Neonatal brain development is associated with cell proliferation and increased protein content. APT and MT offer noninvasive means of characterizing this process through the quantification of protein content and myelination. Applied to the neonatal brain, APT and MT imaging offer effective new tools for the characterization of brain maturation that could enhance our understanding of normal and pathologic brain development.

## Figures and Tables

**Table 1:** The measured APT (%) of frontal lobe deep white matter, basal ganglia, and occipital lobe deep white matter with gestational age (day).

APT (%)				
Case	Gestational age (day)	Frontal lobe deep white matter	Basal ganglia	Occipital lobe deep white matter
1	191	0.2865565	0.5610845	0.2240295
2	196	0.1606295	0.753212	0.531229
3	203	0.314636	0.417282	0.3569482
4	210	0.3283785	1.04580925	0.56413275
5	210	0.658413	1.0398185	0.573494
6	219	0.654974	1.234728333	0.625218
7	219	0.247978	1.27012	0.7158715
8	222	0.5333595	1.270135	0.6364595
9	224	0.62417025	1.0394645	0.87172725
10	229	0.6208725	0.873164	0.6813355
11	233	0.581705	1.2721025	0.65139675
12	237	0.388836	1.23342	0.485775
13	238	0.48234873	1.357225	0.7878892
14	238	0.54149115	1.554525	0.767246
15	240	0.1850855	1.24202	0.58474
16	240	0.8021585	1.54691	0.9852405
17	243	0.43556	1.07769	0.5521545
18	250	0.64959525	1.403605	0.7551365
19	250	0.5394515	1.433305	0.757351
20	252	0.7482053	1.390218167	0.770961333
21	252	0.8250045	1.48694	0.6612495
22	258	0.706682	1.1035045	0.9320605
23	259	0.7196395	1.15165	0.7811165
24	267	0.764964	1.38085075	0.937480875
25	268	0.82505	1.035838	0.896967
26	269	0.6293945	1.650745	1.092922
27	270	0.788212	1.494025	0.8789635
28	275	0.9989034	1.46188	1.085519167
29	277	0.9608	1.52487	1.109135
30	278	1.0568445	1.445595	1.167215
31	279	1.02664408	1.5563758	1.2141181
32	280	0.801769	1.614184	0.97464
33	282	1.1601819	1.53851	1.39712
34	282	1.08711	1.77139	1.28227
35	284	1.0008835	1.491565	1.394685
36	284	1.15272	1.698	1.525505
37	285	1.1803	Null^*∗*^	Null^*∗*^
38	287	1.237795	1.796135	1.558985

Mean ± SD		0.70 ± 0.29	1.30 ± 0.31	0.86 ± 0.32

^*∗*^Null means APT values measurement failed in basal ganglia and occipital lobe due to the presence of partial artifacts of case 37.

**Table 2:** The measured MTR (%) of frontal lobe deep white matter, basal ganglia, and occipital lobe deep white matter with gestational age (day).

MTR (%)				
Case	Gestational age (day)	Frontal lobe deep white matter	Basal ganglia	Occipital lobe deep white matter
1	191	11.0947	16.6731	12.1622
2	196	12.6331	14.9308	10.91905
3	203	10.52812	18.89185	11.2131
4	210	12.6491	19.88995	12.0236
5	210	10.68809	18.02345	12.4753
6	219	12.7266	17.6464	13.0213
7	219	12.85625	17.90015	12.4656
8	222	10.25065	16.3197	11.35305
9	224	10.37185	12.2972	12.6507
10	229	12.55615	13.9513	12.126
11	233	11.926	17.8582	13.89715
12	237	11.00735	16.544	12.1135
13	238	10.09604	17.04255	11.88205
14	238	10.19335	16.16505	11.2817
15	240	11.05615	17.4869	12.04665
16	240	10.772	16.7616	12.2732
17	243	12.57395	19.14885	13.6891
18	250	13.7127	18.099	11.9721
19	250	10.55205	16.5026	12.31035
20	252	10.27066	18.98355	13.0489
21	252	11.41115	20.0092	13.8119
22	258	13.7253	18.84055	13.78625
23	259	12.4354	17.236	11.30005
24	267	12.3934	20.46975	13.4312
25	268	12.86325	15.9051	13.58795
26	269	11.88875	18.43385	13.1562
27	270	12.3402	20.05515	13.46845
28	275	14.5353	23.2817	14.7597
29	277	12.12065	20.6666	14.291
30	278	14.2746	21.6414	14.3113
31	279	12.5789	17.1573	14.66385
32	280	14.5956	20.3918	14.3798
33	282	12.9527	16.2719	14.01905
34	282	12.06405	20.9366	13.9
35	284	12.79625	17.0396	11.9296
36	284	13.1498	22.40645	13.1636
37	285	13.48875	21.6642	14.54245
38	287	11.1343	16.4571	12.86825

Mean ± SD		12.09 ± 1.28	18.16 ± 2.34	12.90 ± 1.09

## Abbreviations

MT: Magnetization transfer
APT: Amide proton transfer
MTR: Magnetization transfer ratio
CEST: Chemical exchange saturation transfer.
